# Validation data of a rabbit antiserum and affinity purified polyclonal antibody against the N-terminus of human GPR30

**DOI:** 10.1016/j.dib.2016.03.054

**Published:** 2016-03-19

**Authors:** Mohan C. Manjegowda, Paridhi Singhal Gupta, Anil M. Limaye

**Affiliations:** Department of Biosciences and Bioengineering, Indian Institute of Technology Guwahati, Guwahati 781039, Assam, India

## Abstract

Rabbit antiserum was generated against the N-terminus of human GPR30 followed by peptide affinity purification. In this article, the methodology used and validation data are presented. The peptide affinity purified polyclonal antibody specifically detects human GPR30 in ELISA and on western blots of total protein prepared from human breast cancer cell lines.

**Specifications Table**

**Table:** 

Subject area	Biology
More specific subject area	GPR30 antibody
Type of data	Graphs, Figures
How data was acquired	ELISA, Western blotting
Data format	Processed
Experimental factors	Immunization of rabbits with N-terminus peptide of human GPR30, collection of antiserum and peptide affinity purification
Experimental features	Validation of the antiserum and peptide affinity purified antibody by ELISA and western blotting
Data source location	Guwahati, Assam, India
Data accessibility	Data presented in this article

**Value of the data**

• The antibody generated can serve as a tool for basic and clinical research in the field of GPR30 biology.

## 1 Data

**Fig. 1:**
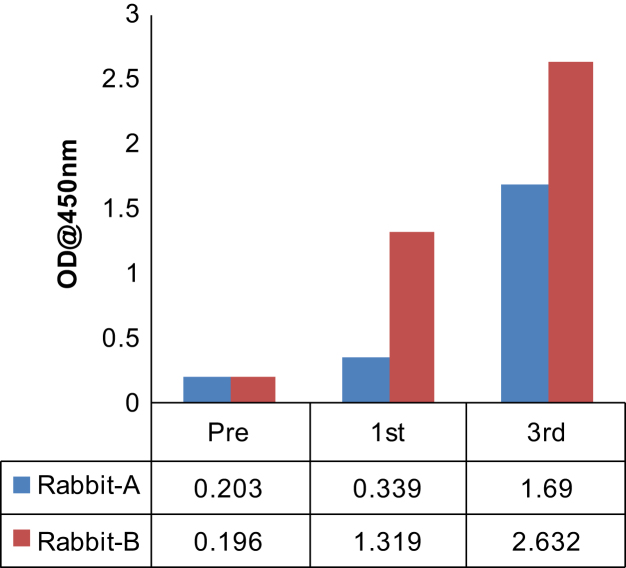
Indirect ELISA for testing the reactivity of immune serum. First and third bleeds of two rabbits (A and B) were compared with their respective pre-immune sera using a protocol described in Materials and reagents (Section 2.4). Immune sera of B were relatively more reactive as compared to those obtained from A. Third bleed of B was most reactive.

**Fig. 2:**
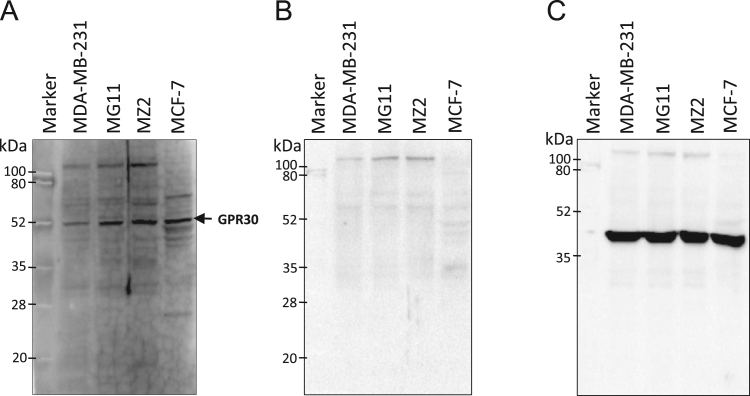
Quality assessment of antiserum against N-terminus of GPR30. Protein lysates prepared from a panel of breast cancer cell lines were fractionated by 10% SDS-PAGE under denaturing conditions and transferred to nitrocellulose membranes. Membranes were subjected to western blotting analysis followed by chemiluminiscence detection. The primary antibodies for each of the above panels are- A. 1 in 1000 dilution of antiserum from Rabbit B (bleed 3); B. No primary antibody; C. 1 in 5000 dilution of commercial anti-β-actin antibody.

**Fig. 3:**
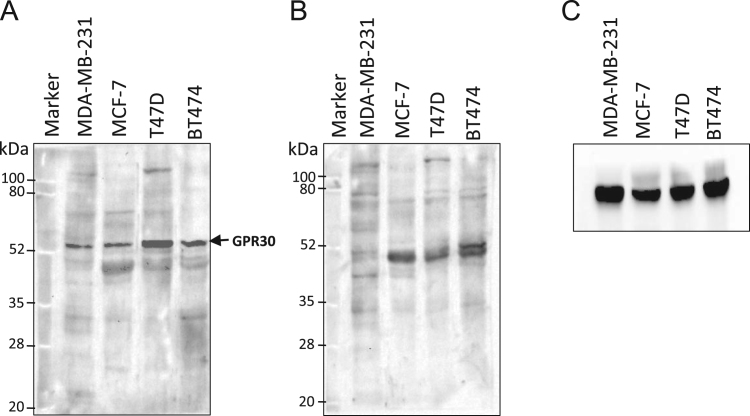
Quality assessment of antiserum against N-terminus of GPR30. Protein lysates prepared from a panel of breast cancer cell lines were fractionated by 10% SDS-PAGE under denaturing conditions and transferred to nitrocellulose membrane. Membranes were subjected to western blotting analysis followed by chemiluminiscence detection. The primary antibodies for each of the above panels are- A. 1 in 10,000 dilution of antiserum from Rabbit B (bleed 3); B. 1 in 10,000 dilution of pre-immune serum from Rabbit B; C. 1 in 5000 dilution of commercial anti-β-actin antibody.

**Fig. 4:**
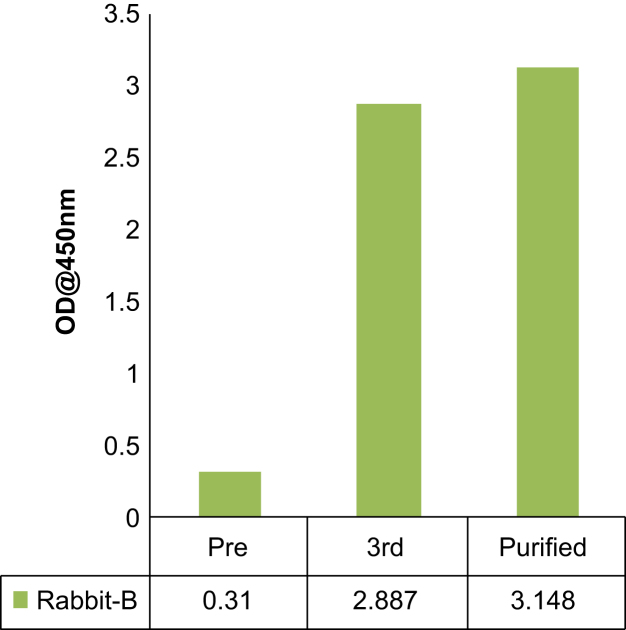
Indirect ELISA for testing the reactivity of the peptide affinity purified antibody. Purified antibody shows similar reactivity as that of the original antiserum (Rabbit-B third bleed).

**Fig. 5:**
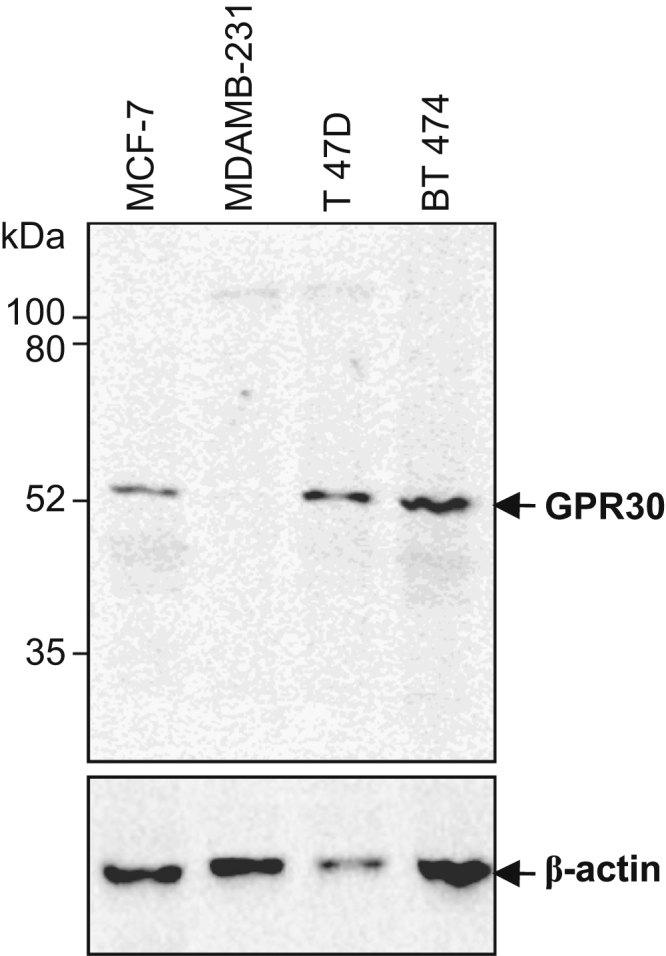
Detection of GPR30 in total protein by affinity purified antibody. Proteins were fractionated by 10% SDS-PAGE under denaturing conditions and transferred to nitrocellulose membranes. Membranes were subjected to western blotting analysis followed by chemiluminiscence detection. The anti-β-actin antibody was used in a dilution of 1:5000 and the affinity purified primary antibody was used in a dilution of 1:15,000.

After immunization of two rabbits (A and B) with the peptide antigen, the antiserum harvested from the third bleed of rabbit B that was collected after the seventh booster, was found to be the most reactive compared to pre-immune serum (Fig. 1). Western blots of total protein prepared from human cell lines with this antiserum resulted in the detection of ~52 kDa band of GPR30 along with other non-specific proteins, which were also detected by pre-immune serum or secondary antibody alone (Fig. 2, Fig. 3). The affinity purified antibody obtained from the antiserum (third bleed) of rabbit B showed similar reactivity to that of the antiserum (Fig. 4). It produced clean western blotting results, in which, only one ~52 kDa band of GPR30 was detected (Fig. 5).

## 2 Experimental design, materials and methods

### 2.1 Experimental design

Rabbits were immunized with N-terminus peptide of human GPR30 and hyperimmune serum was collected after several boosters. Immunoreactivity of the immune serum was checked by indirect ELISA and the antiserum with the highest reactivity was tested for the specificity by western blotting. Upon confirmation of specificity, immunoglobulins were affinity purified and reconfirmed by indirect ELISA and western blotting.

### 2.2 Materials and reagents

Plasticware for cell culture was from Tarsons (Kolkata, India) and Greiner Bio-One (GmbH, Germany). Phenol red-containing media (DMEM and RPMI-1640) and fetal bovine serum (FBS) for cell culture were from Gibco (NY, USA). Radioimmunoprecipitation assay (RIPA) buffer was purchased from Sigma Aldrich (MO, USA) and EDTA-free protease inhibitor cocktail was purchased from TAKARA (CA, USA). Nitrocellulose membrane (0.45 µ) used for western blotting was from Genetix (New Delhi, India). Anti-β-actin mouse polyclonal antibody was purchased from Ambion (Cat. #AM4302). Antibiotics and trypsin-EDTA were purchased from HiMedia (Mumbai, India). All other chemicals and buffers were from SRL (Mumbai, India) or Merck (Mumbai, India).

### 2.3 Generation of polyclonal antibody and affinity purification

Polyclonal antibody generation and peptide affinity purification was performed at Abgenex Pvt. Ltd., Bhubaneswar, India. N-terminus peptide (MDVTSQARGVGLEMYPGTAQPAA) [1] of GPR30 was chemically synthesized with an extra cysteine residue at the C-terminus of the peptide. The peptide was cross-linked to Keyhole Limpet Haemocyanin (KLH, Pierce, Cat. #77600) using maleimide-sulfhydryl chemistry. KLH was activated by treating with sulfosuccinimidyl 4-(N-maleimidomethyl) cyclohexane-1-carboxylate (Sulfo-SMCC, Pierce, Cat. #22322). Maleimide-activated KLH was then purified by gel filtration chromatography and mixed with peptide for cross-linking. Efficiency of conjugation was assessed by determining the free sulfhydryl groups before and after conjugation using Ellman׳s reagent (Pierce, Cat. # 22582). Two rabbits (A and B) were immunized with antigen (KLH conjugated peptide) in Complete Freund׳s Adjuvant (CFA) or Incomplete Freund׳s Adjuvant (IFA) after the collection of pre-immune serum. The first immune serum was collected after primary immunization (200 µg antigen/rabbit in CFA) and five boosters (100 µg antigen/rabbit in IFA). Subsequently, the next two batches (second and third bleeds) of immune sera were collected after 6^th^ and 7^th^ boosters, respectively. First and third batches of immune sera were tested for immune reactivity by indirect ELISA (Fig. 1). Pre-immune serum served as negative control. Amongst the bleeds collected, the third bleed of rabbit B was found to be the most reactive (Fig. 1). The immune serum from third bleed of rabbit B detected the ~52 kDa band of GPR30 (Fig. 2A) which is consistent with other reports [2], [3]. Several non-specific bands were also observed. However, the non-specific bands were also detected on western blots probed only with the secondary antibody (Fig. 2B). As shown in Fig. 3A, a specific ~52 kDa band of GPR30 was detected in breast cancer cell lines along with other non-specific bands which were also detected by the pre-immune serum (Fig. 3B). This immune serum was affinity purified against the immunogenic peptide using the sulfo-link matrix. Immunoreactivity of the affinity purified antibody was confirmed by indirect ELISA (Fig. 4). This antibody detected a single prominent ~52 kDa GPR30 band against a much cleaner background on western blots of total proteins from breast cancer cell lines (Fig. 5).

### 2.4 Indirect ELISA

On day 1, the peptide antigen (200ng/well) was coated on a 96-well plate (Nunc-Immuno plate, Cat. #439454, F96 Cert. MaxiSorp) for 2 h at room temperature followed by overnight incubation at 4 °C. On day 2, the plates were kept on a shaker at room temperature for 2 h. The wells were then washed with phosphate buffered saline containing 0.05% Tween 20 (PBST), followed by blocking with 5% skimmed milk in PBST for 1 h. After three washes with PBST, 100 μL per well of primary antibody (diluted 1:5000 in PBST containing 1% skimmed milk) was added to each well and incubated at room temperature. After 2 h the wells were washed as above. 100 μL of HRP-conjugated secondary antibody (1:5000 diluted in PBST containing 1% skimmed milk) was added to each well and incubated for 1 h. After three washes with PBST, 100 µL of 1X TMB/H_2_O_2_ solution was added and kept in dark for 3–5 min. Thereafter, the 96-well plate was read at 450 nm.

### 2.5 Cell culture

MCF-7 cell line was a kind gift from Dr. Dipak Datta (CDRI, Lucknow, India). T47D and BT474 were gifted by Dr. Prathibha Ranganathan (CHG, Bangalore, India). MDA-MB-231 cells were procured from NCCS, Pune, India. MG11 and MZ2 are derivatives of MDA-MB-231 generated in-house. Cell lines were cultured in a humidified CO_2_ incubator maintained at 37 °C and 5% CO_2_. MCF-7 and BT474 were cultured in DMEM, and T47D, MDAMB-231, MG11 and MZ2 cells were cultured in RPMI-1640 medium. Both the media contained phenol red and were supplemented with 10% FBS, 100 U/mL penicillin, and 100 µg/mL streptomycin.

### 2.6 Western blotting

Cells lysates were prepared in ice-cold RIPA buffer with protease inhibitors. Lysates were collected in pre-chilled 1.5 mL tubes and incubated on ice for 15 min. Cell debris was removed by centrifugation at 15,000×g for 15 min at 4 °C. Supernatants were stored as aliquots of 100 µL in 0.5 mL tubes at −20 °C until use. Total protein was estimated by Lowry׳s method [4]. 30 µg of total protein was resolved by 10% SDS-PAGE and transferred to 0.45 µ nitrocellulose membrane using semi-dry transfer method. Blotting was done either at constant current (140 mA) or voltage (16 V) for 80 min. After the transfer, blots were stained with Ponceau S and scanned. Ponceau S stain was removed by washing in PBS for 5 min followed by a 5 min wash with Tris buffered saline containing 0.05% Tween 20 (TBST). The blots were then blocked in 1% gelatin in TBST for 2 h at room temperature followed by overnight incubation at 4 °C in 0.1% gelatin in TBST. Next day, the blots were brought to room temperature and rinsed with TBST. Blots were then probed with primary antibody diluted in 0.1% gelatin in TBST for 2 h at room temperature. Blots were washed for 30 min with TBST (3×10 min) to remove unbound antibody. Blots were then incubated with HRP-tagged secondary antibody (1:5000 dilution in 0.1% gelatin in TBST) for 1 h followed by three TBST washes of 10 min each. Bands were visualized with enhanced chemiluminescence reagent (Santa Cruz Biotechnology, CA, USA). Chemidoc XRS+ system (BioRad) was used to capture the images.

## References

[1] C.M. Revankar, D.F. Cimino, L.A. Sklar, J.B. Arterburn, E.R. Prossnitz, A transmembrane intracellular estrogen receptor mediates rapid cell signaling., Science (80-.). 307 (2005) 1625–30. 10.1126/science.1106943. 10.1126/science.1106943 15705806

[2] Jala V.R., Radde B.N., Haribabu B., Klinge C.M. (2012). Enhanced expression of G-protein coupled estrogen receptor (GPER/GPR30) in lung cancer. BMC Cancer.

[3] Kolkova Z., Noskova V., Ehinger a, Hansson S., Casslén B. (2010). G protein-coupled estrogen receptor 1 (GPER, GPR 30) in normal human endometrium and early pregnancy decidua. Mol. Hum. Reprod..

[4] Lowry O.H., Rosebrough N.J., Farr A.L., Randall R.J. (1951). Protein measurement with the Folin phenol reagent. J. Biol. Chem..

